# ChatGPT and Artificial Intelligence in Medical Writing: Concerns and Ethical Considerations

**DOI:** 10.7759/cureus.43292

**Published:** 2023-08-10

**Authors:** Alexander S Doyal, David Sender, Monika Nanda, Ricardo A Serrano

**Affiliations:** 1 Anesthesiology, University of North Carolina at Chapel Hill School of Medicine, Chapel Hill, USA

**Keywords:** ai & robotics in healthcare, natural language processing, medical writing, ethics, machine learning, artificial intelligence (ai)

## Abstract

Artificial intelligence (AI) language generation models, such as ChatGPT, have the potential to revolutionize the field of medical writing and other natural language processing (NLP) tasks. It is crucial to consider the ethical concerns that come with their use. These include bias, misinformation, privacy, lack of transparency, job displacement, stifling creativity, plagiarism, authorship, and dependence. Therefore, it is essential to develop strategies to understand and address these concerns. Important techniques include common bias and misinformation detection, ensuring privacy, providing transparency, and being mindful of the impact on employment. The AI-generated text must be critically reviewed by medical experts to validate the output generated by these models before being used in any clinical or medical context. By considering these ethical concerns and taking appropriate measures, we can ensure that the benefits of these powerful tools are maximized while minimizing any potential harm. This article focuses on the implications of AI assistants in medical writing and hopes to provide insight into the perceived rapid rate of technological progression from a historical and ethical perspective.

## Editorial

Introduction

The use of technological advances goes through cycles of both disruptive change and gradual transitions. Communication in particular has seen many iterations of technology, from the printing press to more recent changes such as spell check and algorithms that predict what you might like to say in an email or text. Historically, slow and gradual technological changes have been more easily accepted and well utilized. In contrast, rapid changes in new technologies are often met with resistance. Time, experience, and thoughtful policies are needed in order for society to accept and safely utilize advances in technology. Unfortunately, none of these key elements necessary to utilize new technology are present in our current artificial intelligence (AI) environment.

Recently, AI reached a critical stage of development where a non-expert could utilize the technology with little or no computer coding background or specialized medical knowledge. Given how rapidly the field of adaptive AI is evolving, this raises serious concerns. We will discuss the role of generative models in medical writing, with a focus on the historical roots of AI and the ethical implications of its current trajectory.

History

The evolution of word processing AI, also known as AI-assisted writing, has been driven by advances in natural language processing (NLP) and machine learning (ML) technologies. NLP refers to the branch of computer science that involves understanding written text by combining computational linguistics (rule-based language modeling, e.g., grammar) with statistical/machine-learning/deep learning models. Ideally, NLP allows computers to ‘understand’ human written or spoken language. The history of word processing AI can be broken down into several key phases [1-3].

In the history of word processing programs and AI integration, several distinct phases have shaped the evolution of these technologies. During the early phase of the 1980s-1990s, programs like Microsoft Word and WordPerfect primarily focused on basic editing and formatting functionalities.

In the 1990s-2000s, the introduction of grammar checkers marked a significant step forward. Word processing programs started incorporating rule-based algorithms to identify and correct grammar and spelling errors. This reduced the effort needed for basic editing, thereby freeing up the writer to focus on his/her ideas. 

As technology progressed further, the 2000s-2010s witnessed the integration of predictive text capabilities into word processing programs. By utilizing statistical models like n-gram models, these systems suggested words and phrases to users as they typed, improving writing efficiency. This marked a departure from grammar and spelling basics. Now, communication technology has begun to anticipate and predict basic phraseology, leaving the overall structure and development of the topic to the writer.

The 2010s-2020s brought about groundbreaking advancements in deep learning, a subset of machine learning. Word processing AI benefited significantly from this technology, employing neural networks with multiple layers, mimicking the human brain's learning mechanism. Large datasets trained language models like GPT-3 and GPT-4 to generate highly coherent and natural-sounding text. These models found use in various tasks, including grammar checking, text summarization, and question answering.

As AI technology continued to advance, the 2020s to the present day saw the rise of AI-assisted writing tools. Based on deep learning models, these tools provide valuable suggestions on grammar and writing style and even assist with plot and character development for creative writing. Many popular writing software programs, such as Grammarly, Hemingway, and ProWritingAid, have integrated these AI-assisted features, making them widely accessible to users seeking enhanced writing support.

Overall, the evolution of word processing AI has been marked by a gradual increase in the sophistication and intelligence of the underlying technology, leading to more powerful and versatile tools for writers and other users [4-6]. The three major movements towards our current moment with AI can be described as word-based editing, sentence- or phrase-based editing, and idea synthesis. This last leap forward represents a qualitative difference in the kind of work we are asking of AI and ourselves. At this juncture, the author is only responsible for the initial idea. This reality distills the power of the old tech adage, "garbage in, garbage out."

Current AI

Several forms of AI have been used for writing, each with its own strengths and weaknesses. One form is Natural Language Generation (NLG) systems, which use AI to generate written text in a human-like style automatically. These systems are beneficial for tasks such as generating reports and summaries. Another form is ML-based text generation models that use statistical techniques to generate text based on patterns in a training dataset. These models are commonly used to create news articles, product descriptions, and other types of written content [3].

Neural network-based models, such as GPT, BERT, and others, are also popular for text generation. These models use deep learning to generate text that is often indistinguishable from human-written text. They have been used in many applications, such as chatbots, language translation, and more. Rule-based systems, on the other hand, use a set of predefined rules to generate text based on a specific set of inputs. These systems are typically used in applications with highly structured output, such as generating code or legal documents [7].

Hybrid models, which combine the above methods to generate text with a high degree of accuracy and naturalness, are also being increasingly used. These models combine the ability of rule-based systems to generate structured text with the power of neural network-based models to generate natural text. Finally, AI-assisted writing tools are software that helps writers write better by providing suggestions, grammar checking, and more. These tools are particularly useful for writers who want to improve their writing skills or for people who are not native speakers of the language they are writing in [8].

ChatGPT is the newest generation of artificial intelligence assisting the writing process. ChatGPT is a language generation model developed by OpenAI. It is based on the GPT (generative pre-training transformer) architecture, which uses deep learning to generate human-like text. ChatGPT is trained on a large dataset of conversational text and can create responses to user input in a conversational context. It can be used for various natural language processing tasks such as language translation, text summarization, and question answering [9].

Citation issues

It is clear that artificial intelligence writing systems such as ChatGPT are here to stay, and different platforms are likely to be developed in the future. One must ask, should these artificial intelligence writing systems be used for medical writing? [5,6]. And if so, how should they be cited in the medical literature [10,11]?

Regarding authorship, it may be appropriate to credit ChatGPT as a tool or resource used in the research process rather than listing it as an author. Authorship is generally reserved for individuals who have made a significant intellectual contribution to the work, such as designing and conducting the study, analyzing the data, and writing the manuscript. However, it is always good practice to acknowledge the tools and resources that were used in the research and writing process, such as the use of AI and machine learning tools [11,12].

It is essential to be transparent about the use of language generation models in research, as this allows others to understand the potential limitations and biases of the generated text and to replicate the research if needed. Additionally, proper citation of the model also gives credit to the model's creators and the training data's contributors [10].

Medical writing

ChatGPT and other language generation models based on deep learning techniques, such as GPT-3, can be used for various natural language processing tasks, including medical writing. However, it is essential to note that using AI-generated text in the medical field requires careful consideration and review by medical experts to ensure the accuracy and reliability of the generated text [13].

Some suggested uses of ChatGPT and other language generation models in medical writing include generating reports and summaries of medical research papers and clinical trials, creating patient-specific medical information like discharge summaries and patient education materials, assisting in the writing of medical textbooks and guidelines, generating product labels and package inserts for medical devices and drugs, creating a chatbot or virtual assistant capable of answering medical-related questions, and assisting in highly protocolized letter writing, such as preauthorization letters to insurance companies, work excuses, or letters of recommendation.

Although useful in these contexts, it is necessary for clinicians to critically review and validate any computer-generated text before it is used in any clinical setting or research. AI-generated text has the potential to perpetuate bias, misinformation, and plagiarism. Additionally, as the field of medicine is constantly evolving, computer models should be retrained regularly to ensure they stay up-to-date with the latest knowledge.

Ethical and other considerations

ChatGPT, a language generation model developed by OpenAI, is a powerful tool that can be used for various natural language processing tasks, including medical writing. However, its use also raises significant ethical concerns that must be carefully considered, including bias, misinformation, privacy, a lack of transparency, and plagiarism [13-15]. 

One primary point of interest is bias. Language generation models are trained on large datasets of text, and any biases present in the training data may be reflected in the generated text. This can lead to discriminatory or offensive language, perpetuating harmful stereotypes. For example, if a model is trained on a dataset that contains a disproportionate amount of text written by men, it may generate text that reflects a male-centric perspective. If a model is trained on a dataset containing "fake news," it will produce consistently inaccurate text [7].

Therefore, measures to prevent bias in generative AI models should be put in place in a prospective manner instead of a retrospective manner. ChatGPT's initial development stage consisted of scraping hundreds of billions of words from the internet with insufficient attention to filtering out toxic themes and bias. It is very difficult for a deployed model to correct biased outputs once it has been trained. Paradoxically, any attempts to improve data by limiting sources that the AI is incorporating will, in fact, produce their own set of biases [16].

Another problem is misinformation. Language generation models can generate text that is not factually accurate, which can be a concern when the generated text is used in sensitive domains such as medicine or finance. For example, if a model generates text that provides incorrect medical information, it could potentially harm patients [15]. Further, these models often present information in an authoritative tone of voice without having actual expertise. Although efficient in producing vague general knowledge, it is insufficient when generating information at the subspecialist level.

Privacy is also a significant concern. Language generation models can be used to generate highly personalized text, such as patient-specific medical information. This patient-specific information requires the AI to have access to a patient’s protected medical record or medical data. There exists a high potential to harm patient privacy rights and erode the faith that patients and clinicians may have entrusted in AI language generation models. Mistrust in these systems may hinder their successful integration into clinical practice.

Lack of transparency is also problematic. Language generation models can be challenging to understand, and it can be hard to know how a specific output is generated. This can make it difficult to determine the quality of the generated text or to identify and correct any errors. Additionally, the sources used by the AI writers are not readily apparent, and it is possible that non-peer-reviewed medical literature is being used to create content [5].

Even more malicious is the use of made-up scientific references containing misinformation, which could contaminate our existing biomedical knowledge databases at scale. Open AI is attempting to implement a watermark feature that labels content created by ChatGPT [17]. Other detection tools, such as DetectGPT, are in development. DetectGPT has been reported to correctly determine authorship in 95% of test cases [18].

Besides ethical considerations, another concern when using ChatGPT and other language generation models for creative writing is the potential to stifle creativity and originality. Similarly, spell checkers have trivialized our educational efforts to learn proper spelling. The generated text may be highly polished and grammatically correct, but it may lack the individuality and creativity that are often associated with human-written text. This could lead to a homogenization of written content, where all text starts to sound the same [19].

Yet another matter is the potential for the model to generate text that plagiarizes existing work. Language generation models are trained on a vast amount of text, and the model may unintentionally generate text similar to or identical to existing work. This could lead to legal issues and ethical concerns [20,21].

Moreover, the creative process is about the final product and the journey of creating something new, utilizing the writer's personal experience and perspective. Using AI-generated text may take away from the personal and emotional investment of the writer in the writing process, which can be an important aspect of the creative process [22].

Job displacement is another worry. Automated text generation can be used to automate tasks that were previously done by human medical writers, editors, and others. This could lead to job displacement and economic disruption. Furthermore, using AI-generated text can also lead to a dependence on the technology, which can be harmful if the models are unavailable or fail [23].

In conclusion, while ChatGPT and other language generation models have the potential to revolutionize the field of medical writing and other natural language processing tasks, it is crucial to consider the ethical concerns that come with their use. These include bias, misinformation, privacy, lack of transparency, job displacement, stifling creativity, plagiarism, authorship, and dependence. Therefore, it is essential to develop strategies to address these concerns, such as common bias and misinformation detection, ensuring privacy, providing transparency, and being mindful of the impact on employment. Experts should review and validate the output generated by these models before they are used in any clinical or medical context. By considering these ethical concerns and taking appropriate measures, we can ensure that the benefits of these powerful tools are maximized while minimizing any potential harm [1,14].

Summary

In conclusion, while AI-generated text can offer numerous benefits and enhance various aspects of medical writing, we must approach its use with great caution and mindfulness. The advantages of efficiency, productivity, and support in generating content must be weighed against potential downsides like bias, misinformation, plagiarism, and privacy concerns. As AI technologies continue to advance rapidly, it is essential for the medical community, policymakers, and society as a whole to continually grapple with the ethical implications and challenges posed by AI-generated text.

The responsible use of AI in medical writing necessitates clear guidelines, robust validation processes, and close collaboration between AI systems and human expertise. Transparency and acknowledgment of the role of AI in generating text are vital to ensuring that human authors remain accountable for the final output. Additionally, ongoing research and development are required to address bias detection, misinformation prevention, and privacy protection in AI-generated text.

Ultimately, the question of whether we should use AI-generated text in medical writing will persist. The answer lies in our ability to strike a delicate balance between leveraging AI's potential while respecting the importance of human creativity, critical thinking, and ethical considerations. As we navigate this evolving landscape, it is crucial to maintain a thoughtful approach and prioritize the well-being of patients, the integrity of medical knowledge, and the overall advancement of healthcare practices. By doing so, we can harness the power of AI while upholding the highest standards of medical writing and patient care.
